# Cross-sectional associations between the types/amounts of beverages consumed and the glycemia status: The Japan Public Health Center-based Prospective Diabetes study

**DOI:** 10.1016/j.metop.2022.100185

**Published:** 2022-04-20

**Authors:** Yusuke Kabeya, Atsushi Goto, Masayuki Kato, Yoshihiko Takahashi, Akihiro Isogawa, Yumi Matsushita, Tetsuya Mizoue, Manami Inoue, Norie Sawada, Takashi Kadowaki, Shoichiro Tsugane, Mitsuhiko Noda

**Affiliations:** a Sowa Clinic, Kanagawa, Japan; b Epidemiology and Prevention Group, Center for Public Health Sciences, National Cancer Center, Tokyo, Japan; c Department of Health Data Science, Graduate School of Data Science, Yokohama City University, Kanagawa, Japan; d Health Management Center and Diagnostic Imaging Center, Toranomon Hospital, Tokyo, Japan; e Division of Diabetes and Metabolism, Iwate Medical University School of Medicine, Iwate, Japan; f Department of Internal Medicine, Mitsui Memorial Hospital, Tokyo, Japan; g Department of Clinical Research, Center for Clinical Sciences, National Center for Global Health and Medicine, Tokyo, Japan; h Department of Epidemiology and Prevention, Center for Clinical Sciences, National Center for Global Health and Medicine, Tokyo, Japan; i Department of Diabetes and Metabolic Diseases, The University of Tokyo, Tokyo, Japan; j Toranomon Hospital, Tokyo, Japan; k Department of Diabetes, Metabolism and Endocrinology, Ichikawa Hospital, International University of Health and Welfare, Chiba, Japan

**Keywords:** Diabetes mellitus, Fasting plasma glucose, Glycated hemoglobin, Beverage consumption, The JPHC Diabetes study

## Abstract

**Background:**

The associations between the types/amounts of beverages consumed in daily life and measures of the glycemia status were investigated in a Japanese population-based cohort.

**Methods:**

Data from the baseline survey of the Japan Public Health Center-based Prospective Diabetes cohort were used. A cross-sectional analysis was performed in 3852 men and 6003 women who were evaluated under the fasting condition. The daily consumptions of coffee, green tea, oolong tea, black tea, soft drinks, fruit juices, or plain water were assessed using a self-reported questionnaire. Multivariable-adjusted linear regression analyses were performed using measures of the glycemia status (fasting plasma glucose (FPG) and glycated hemoglobin (HbA1c) ) as dependent variables and the types/amounts of beverages consumed as the independent variables, to calculate the differences according to the types/amounts of beverages consumed.

**Results:**

In the multivariable-adjusted models, coffee consumption of ≥240 mL/day was significantly associated with a change of the FPG level by −1.9 mg/dL in men (p = 0.013) and −1.4 mg/dL in women (p = 0.015), as compared to coffee consumption of 0 mL/day. No significant association of the FPG level was observed with any of the other types/amounts of beverages consumed. On the other hand, significant associations were found between the HbA1c levels and consumption of several types of beverages.

**Conclusions:**

High coffee consumption was associated with lower FPG levels in this Japanese population. Some unexpected associations of the HbA1c levels with the consumption of some types of beverages were observed, which need to be further investigated.

## Abbreviations:

BMI: body mass index
FPG: fasting plasma glucose
HbA1c: glycated hemoglobin
JDS: Japan Diabetes Society
JPHC: Japan Public Health Center-based Prospective
PHC: public health center

## 1 Introduction

Diabetes mellitus is among the most prevalent of chronic diseases worldwide. Currently, the epicenter of the disease endemic has shifted from Western to Asian countries, including Japan, because of the large populations and dramatic changes in lifestyles, including the dietary patterns and physical activity levels [1]. In line with the known association of the risk of diabetes with dietary habits, beverage consumption is also reported to be linked to the risk of diabetes. Favorable and unfavorable effects of several types of beverages on the glucose metabolism have been reported. Coffee, which is commonly consumed worldwide, could have a protective effect against the development of diabetes [2], while consumption of sweetened beverages could increase the risk of obesity and diabetes [3,4]. As for other types of beverages, such as green tea [[5], [6], [7], [8], [9], [10]], oolong tea [8,11], black tea [8,12], and plain water [[13], [14], [15], [16]], the evidence remains scarce or controversial. A look at the daily lives of the general population would show that people consume a variety of beverages, and it may be difficult to select any one beverage as the beverage of choice. Consumption of certain types of beverages could be closely linked to that of other types of beverages, which would make it difficult to disentangle the independent effects of each of the beverages on the glucose metabolism. This could explain why only a few studies until now have comprehensively collected data on the beverage consumption of subjects and analyzed the associations between the types of beverages consumed and the glucose metabolism.

In the present study, we investigated the associations between the types/amounts of beverages consumed by the subjects and measures of the glycemia status (hereinafter, glycemic measures) in the Japan Public Health Center-based Prospective (JPHC) Diabetes cohort, which consists of registered inhabitants of areas served by 10 public health centers (PHCs) across Japan.

## 2 Subjects, materials and methods

### 2.1 Study population and study design

The present study was based on the data obtained from research on diabetes performed in a large cohort study in Japan, namely, the Japan Public Health Center-based prospective Study (JPHC study). The JPHC study was initiated in 1990 for Cohort I, with Cohort II added in 1993. Cohort I consists of all registered Japanese inhabitants aged 40–59 years old living in areas of Japan served by 11 PHCs in Cohort I and Cohort II consists of all Japanese inhabitants aged 40–69 years old living in the same aforementioned areas at the start of each survey. The details of the study are described elsewhere [17]. The diabetes study (JPHC diabetes study) was performed in the participants of the JPHC study living in areas covered by 10 of the PHCs. Among the registered inhabitants of the JPHC study, those who underwent annual health checkups in each PHC area were recruited. A self-reported questionnaire specific to diabetes and lifestyle, and measurement of the glycated hemoglobin (HbA1c) were added to their routine health checkup examinations. The initial survey of the JPHC diabetes study was performed in 1998–1999 for Cohort II and in 2000 for Cohort I. The 5-year follow-up survey was performed in the same way in 2003–2004 for Cohort II and in 2005 for Cohort I. For the present study, data of the participants of the initial survey were included. Of the 28,363 participants who responded to the questionnaire of the initial survey of the diabetes study, 3668 participants with missing data for any of the exposure variables described later were excluded. In addition, 1025 participants who reported having been diagnosed as having diabetes or were taking medications for diabetes were also excluded, as these factors could also influence the fasting plasma glucose (FPG) and HbA1c levels. Of the remaining 23,670 participants, the 9855 participants who had been examined under the fasting condition were included in the analysis. The participants provided their written informed consent for participation in this study. The study was conducted with the approval of the ethics committee of the International Medical Center of Japan, the former name of the National Center for Global Health and Medicine, and the ethics committee of Saitama Medical University.

### 2.2 Data collection

A self-administered questionnaire was completed by each of the participants at the 5-year and/or 10-year follow-up of the JPHC study, which contained questions about previously diagnosed medical conditions, medications taken, and lifestyle factors such as the beverages consumed, physical activity levels, smoking, alcohol intake, and employment status. In addition, a separate questionnaire for the JPHC diabetes study was also completed, which contained questions about the detailed past history of diabetes, treatment of diabetes, family history of diabetes, and daily time spent walking. Both data from the JPHC study questionnaire administered upon entry into the JPHC diabetes study and the JPHC diabetes study questionnaire were used for the present study.

### 2.3 Measurements of beverage consumption

The JPHC questionnaire included a question on the frequency of beverage consumption with the following choices: 0, 1–2, 3–4, or 5–6 times/week and 1, 2–3, 4–6, 7–9, or ≥10 cups/day. The types of beverages included in the question were two forms of green tea (Sencha and Bancha/Genmaicha), oolong tea, black tea, two types of coffee (coffee excluding canned coffee and canned coffee), two types of fruit juices (apple juice and orange juice), two types of soft drinks (cola and energy drink), and two types of plain water (tap water and bottled water). In regard to coffee and tea consumption, the subjects were also asked about the daily number of teaspoonfuls of sugar that they added to the coffee or tea. The amount of consumption of each beverage (mL/day) was calculated by multiplying the frequency by the portion size (120 mL/cup for Sencha, Bancha/Genmaicha, oolong tea, black tea, and coffee, 200 mL/cup for apple juice, orange juice, tap water, and bottled water, 250 mL/cup for canned coffee, colas, and energy drinks). For each beverage, the total amount consumed daily was calculated by summing the amounts of the two types of the beverage (listed above) consumed. Details of validation of the method for assessing beverage consumption in the JPHC study are reported elsewhere [18]. In brief, the validity was evaluated by comparing the data with the dietary records for 28 or 14 days. The correlation coefficients obtained from comparisons between the dietary records and the questionnaire responses were as follows: for green tea: 0.44 (men) and 0.53 (women); for coffee: 0.75 (men) and 0.80 (women); for oolong tea: 0.26 (men) and 0.38 (women); for black tea: 0.45 (men) and 0.54 (women); for fruit juice: 0.22 (men) and 0.31 (women); for soft drinks: 0.35 (men) and 0.41 (women) [18].

We divided the participants into four categories according to the beverage consumed, such that the numbers of participants in the different categories were as close as possible. Except for Sencha, the category of 0 mL/day consumption was also set for each beverage. For beverages for which more than half of the participants reported no consumption (0 mL/day), we divided the participants into three categories.

### 2.4 Measurement of the plasma glucose and HbA1c levels

Blood samples collected ≥8 h after the last caloric intake were defined as fasting blood samples. Efforts to standardize the plasma glucose levels measured at the laboratories in the different PHC areas were made by the standardization committee of the JPHC study. The standardization method is reported elsewhere [19] and the accuracy of the measurements has been reported to be satisfactory [19]. As for the HbA1c measurement also, details about the standardization procedure are described elsewhere [20]. In brief, standard samples were provided to each PHC at the time of the initial survey and at the time of the 5-year follow-up survey. The calibration procedure was conducted using the standard samples. The original standard samples were examined and approved by the Japan Diabetes Society (JDS). The procedure for HbA1c calibration used by the JDS is described elsewhere [21]. The HbA1c data were converted to equivalent values of the National Glycohemoglobin Standardization Program, according to a statement issued by the JDS [22].

### 2.5 Other characteristics of the study participants

Information on the characteristics of the study participants were obtained from the health checkup data and responses to the self-administered questionnaire. Body mass index (BMI) was calculated as the weight (in kilograms) divided by the height (in meters) squared, which were measured during the health checkup as part of the JPHC diabetes study. Blood pressure was measured in the right arm by trained nurses using mercury sphygmomanometers during the health checkup. The measurement was performed after the subject had rested for at least 5 min in the sitting position. Hypertension was defined if any of the following criteria were met at the baseline: 1) systolic blood pressure ≥140 mm Hg and/or diastolic blood pressure ≥90 mm Hg; 2) self-reported hypertension; 3) receiving antihypertensive medication(s). Participants were categorized into three groups according to their smoking history (never smoker, past smoker, current smoker). Patients were classified according to their alcohol intake habit as non-drinkers (consumed alcoholic beverages less than once a week) or into one of the three tertiles of the weekly alcohol intake level. Family history of diabetes was defined as the presence of at least one first-degree relative with diabetes. Employment status was classified into two groups (employed, unemployed (including housewives)). As for leisure-time physical activity, participants were considered as physically active if they engaged in sports activities at least 1 day of the week. The daily time spent walking was categorized as follows: <30 min, 30 min to <1 h, 1 h to <2 h, and ≥2 h).

### 2.6 Statistical analysis

We conducted a cross-sectional analysis to examine the associations of the types and amounts of beverages consumed by the subjects and the FPG and HbA1c levels. The analyses were performed separately for the male and female subjects. We first examined the correlations among the type/amount of beverage consumed, the participant characteristics, and glycemic measures using the Spearman rank correlation tests. Then, multivariable-adjusted linear regression analyses were performed using glycemic measures (FPG and HbA1c) as the dependent variables and the beverages consumed as the independent variables. Adjusted means and differences with p values according to the beverages consumed were estimated. In Model 1, the estimates were adjusted for age (continuous), sex, and PHC area. In Model 2, further adjustment was performed for BMI (continuous), smoking status (never, former, current smoker), alcohol intake (non-drinker, tertiles of weekly alcohol intake), family history of diabetes (yes, no), leisure-time physical activity (<1 day/week, ≥1 day/week), daily time spent walking (<30 min, 30 min to <1 h, 1 h to <2 h, ≥2 h), employment status (employed, unemployed), and hypertension (yes, no). In Model 3, the beverages consumed except the examining variable and the daily number of teaspoonfuls of sugar that the subjects added to their tea or coffee were entered into Model 2. The linear trends of the beverage consumption categories were also examined. A mean value of beverage consumption was calculated and assigned to each category. Then, the p values were examined by including the variable as a continuous variable in the models. Statistical analyses were performed using STATA software version 11 (StataCorp, College Station, TX, USA). All statistical tests were two-sided, and p values of less than 0.05 were considered as being statistically signiﬁcant.

## 3 Results

Table 1 shows the baseline characteristics of the study participants. In men (n = 3852, mean age 61.7 years), the mean FPG and HbA1c levels were 99.8 mg/dL and 5.54%, respectively. In women (n = 6003, mean age 61.1 years), the levels were 95.7 mg/dL and 5.49%, respectively. The correlations among the beverages consumed, participant characteristics, and glycemic measures are demonstrated in Table 2. In both sexes, coffee consumption was negatively correlated with the FPG levels. Fruit juice and soft drink consumptions were negatively correlated with the HbA1c levels. In men alone, a negative correlation was observed between water consumption and the HbA1c level. Correlation was also observed between the age and type of beverage consumed. Older participants showed higher consumption of green tea, and lower consumptions of coffee, oolong tea and black tea. In regard to the correlations among the types of beverages consumed, consumptions of beverages other than green tea were positively correlated with each other, while green tea consumption was negatively correlated with soft drink consumption in both men and women. In women alone, green tea consumption showed a positive correlation with black tea consumption and negative correlation with oolong tea consumption.

**Table 1:** Baseline characteristics of the study participants by sex.

		Men (n = 3852)		Women (n = 6003)	
Fasting plasma glucose (mg/dL)		99.8	(14.4)	95.7	(13.0)
Hemoglobin A1c (%)		5.54	(0.55)	5.49	(0.48)
Age, years		61.7	(6.9)	61.1	(6.6)
Body mass index, kg/m^2^		23.6	(2.9)	23.7	(3.2)
Family history of diabetes, yes n (%)		418	(10.9)	794	(13.2)
Smoking status, n (%)	Never	1459	(37.9)	5764	(96.0)
	Past	1082	(28.1)	62	(1.0)
	Current	1311	(34.0)	177	(3.0)
Alcohol intake, n (%)	Non-drinker	2706	(70.3)	5782	(96.3)
	1st half	676	(17.6)	114	(1.9)
	2nd half	470	(12.2)	107	(1.8)
Leisure-time physial activity, active n (%)		922	(23.9)	1818	(30.3)
Daily time spent walking (hours/day)	<0.5	666	(17.3)	969	(16.1)
	≥0.5 and < 1.0	975	(25.3)	1503	(25.0)
	≥1.0 and < 2.0	732	(19.0)	1284	(21.4)
	≥2.0	1479	(38.4)	2247	(37.4)
Employed, yes n (%)		3381	(87.8)	2596	(43.3)
Hypertension, yes n (%)		1725	(44.8)	2286	(38.1)

Values are the mean (standard deviation) or n (%).

**Table 2:** Spearman correlation coefficients, stratified by sex, relating exposure and outcome variables.

	Coffee	Green tea	Oolong tea	Black tea	Fruit juice	Soft drink	Water
Men (n = 3852)							
Fasting plasma glucose	^a^−0.116	0.019	−0.002	−0.002	^c^−0.040	^c^−0.035	0.005
HbA1c	0.011	0.030	−0.025	−0.018	^a^−0.069	^a^−0.073	^a^−0.064
Age	^a^−0.181	^a^0.116	^a^−0.141	^c^−0.033	0.021	^b^−0.044	^c^0.033
BMI	−0.027	^b^−0.047	^a^0.147	^b^0.051	0.019	0.011	^a^0.078
Alcohol intake	0.021	−0.014	0.038	−0.001	^a^0.087	^a^0.148	^a^0.153
Time spent walking	0.026	−0.027	−0.006	−0.017	^b^0.040	^a^0.100	^a^0.084
Coffee							
Grean tea	^a^−0.081						
Oolong tea	^a^0.149	−0.030					
Black tea	^a^0.144	0.020	^a^0.297				
Fruit juice	^a^0.119	0.030	^a^0.243	^a^0.243			
Soft drink	^a^0.236	^b^−0.044	^a^0.216	^a^0.163	^a^0.293		
Water	^a^0.071	0.012	^a^0.149	^a^0.086	^a^0.170	^a^0.157	

Women (n = 6003)							
Fasting plasma glucose	^a^−0.114	^a^0.086	0.000	−0.016	0.017	0.000	^b^0.035
HbA1c	^a^−0.070	^c^0.029	0.001	−0.006	^a^−0.056	^a^−0.061	0.002
Age	^a^−0.248	^a^0.090	^a^−0.175	^a^−0.147	−0.021	^c^−0.031	0.018
BMI	−0.011	^b^−0.040	^a^0.110	^b^−0.034	0.016	^a^0.059	^a^0.103
Alcohol intake	^a^0.048	−0.007	^a^0.053	^b^0.039	0.020	^a^0.047	^c^0.028
Time spent walking	0.013	^c^−0.026	0.021	−0.017	^a^0.055	^a^0.091	^a^0.060
Coffee							
Grean tea	^a^−0.123						
Oolong tea	^a^0.143	^b^−0.027					
Black tea	^a^0.143	^b^0.036	^a^0.262				
Fruit juice	^a^0.079	0.016	^a^0.188	^a^0.177			
Soft drink	^a^0.126	^a^−0.082	^a^0.158	^a^0.101	^a^0.232		
Water	^a^0.070	−0.022	^a^0.140	^a^0.069	^a^0.153	^a^0.131	

^a^P<0.001.

^b^P < 0.01.

^c^P < 0.05.

In the linear regression models used to examine the associations between the types/amounts of beverages consumed and the FPG levels, only coffee consumption was significantly associated with the FPG level (Table 3 and Table 4). In the multivariable-adjusted model (Model 3) in men, coffee consumption of ≥240 mL/day was associated with a change of the FPG level by −1.9 mg/dL (p = 0.013) as compared to coffee consumption of 0 mL/day. In women, coffee consumption of ≥240 mL/day was associated with a change of the FPG level by −1.4 mg/dL (p = 0.015) as compared to coffee consumption of 0 mL/day. A dose-response relationship was also observed in both the male and female subjects (P _*for trend*_ in men = 0.029 and P _*for trend*_ in women = 0.032).

**Table 3:** Fasting plasma glucose and the types/amounts of beverages consumed in men.

Type of beverage	Daily consumption (mL)	Men (n = 3852)									
		n	Model 1			Model 2			Model 3		
			Adjusted mean	Difference	P-value	Adjusted mean	Difference	P-value	Adjusted mean	Difference	P-value
Coffee	0	1160	101.4	ref		101.1	ref		100.8	ref	
	0> and <120	1072	100.1	−1.3	0.032	99.9	−1.2	0.035	100.0	−0.7	0.301
	≥120 and < 240	765	98.8	−2.6	<0.001	98.8	−2.3	<0.001	98.9	−1.8	0.014
	≥240	855	98.1	−3.3	<0.001	98.8	−2.3	0.001	98.9	−1.9	0.013
			Ptrend =	<0.001		Ptrend =	0.002		Ptrend =	0.029	

Green tea	<240	1070	100.4	ref		100.2	ref		100.1	ref	
	≥240 and < 480	964	99.7	−0.7	0.261	99.7	−0.4	0.502	99.8	−0.3	0.634
	≥480 and < 720	937	99.6	−0.8	0.261	99.6	−0.5	0.418	99.7	−0.4	0.483
	≥720	881	99.3	−1.1	0.089	99.5	−0.6	0.326	99.5	−0.6	0.336
			Ptrend =	0.123		Ptrend =	0.374		Ptrend =	0.355	

Oolong tea	0	2362	99.7	ref		100.1	ref		100.0	ref	
	0> and <60	770	99.2	−0.5	0.364	99.0	−1.0	0.078	99.2	−0.7	0.250
	≥60	720	100.6	0.8	0.193	99.7	−0.4	0.526	99.8	−0.1	0.821
			Ptrend =	0.151		Ptrend =	0.659		Ptrend =	0.979	

Black tea	0	3047	99.8	ref		99.9	ref		99.7	ref	
	0> and <60	561	99.6	−0.2	0.736	99.5	−0.4	0.566	100.1	0.3	0.633
	≥60	244	100.1	0.3	0.740	99.4	−0.5	0.599	99.9	0.2	0.821
			Ptrend =	0.814		Ptrend =	0.528		Ptrend =	0.768	

Fruit juice	0	2544	100.0	ref		100.0	ref		99.9	ref	
	0> and <100	996	99.5	−0.5	0.402	99.3	−0.7	0.171	99.5	−0.4	0.450
	≥100	312	99.3	−0.6	0.479	99.8	−0.2	0.774	99.9	0.0	0.983
			Ptrend =	0.356		Ptrend =	0.449		Ptrend =	0.820	

Soft drink	0	2071	100.1	ref		100.0	ref		99.7	ref	
	0> and <100	1057	99.1	−1.1	0.057	99.3	−0.7	0.220	99.5	−0.2	0.715
	≥100	724	99.9	−0.2	0.706	100.0	0.1	0.887	100.4	0.7	0.294
			Ptrend =	0.691		Ptrend =	0.356		Ptrend =	0.279	

Water	0	809	99.8	ref		100.3	ref		100.0	ref	
	0> and <200	610	99.8	0.0	0.971	100.1	−0.2	0.799	100.2	0.2	0.827
	≥200 and < 500	921	99.9	0.1	0.829	100.0	−0.3	0.616	100.1	0.1	0.939
	≥500	1512	99.7	−0.1	0.826	99.3	−1.0	0.104	99.3	−0.7	0.258
			Ptrend =	0.705		Ptrend =	0.070		Ptrend =	0.107	

Model 1: Adjusted for age (continuous) and PHC areas.

Model 2: Model 1 + adjusted for BMI (continuous), smoking status (never, former or current smoker), alcohol intake (non-drinker or tertiles of alcohol intake), family history of diabetes (yes or no), leisure-time physical activity (<1 day/week or ≥ 1 day/week), daily time spent walking (<30 min, 30 min to less than 1 h, 1 h to less than 2 h, or 2 h or more), employment status (employed or unemployed) and hypertension (yes or no).

Model 3: Model 2 + adjusted for the daily amount of teaspoons of sugar used in tea or coffee + respective beverage consumption except the variable of interest.

**Table 4:** Fasting plasma glucose and the types/amounts of beverages consumed in women.

Type of beverage	Daily consumption (mL)	n	Women (n = 6003)								
			Model 1			Model 2			Model 3		
			Adjusted mean	Difference	P-value	Adjusted mean	Difference	P-value	Adjusted mean	Difference	P-value
Coffee	0	1984	96.8	ref		96.6	ref		96.4	ref	
	0> and <120	1920	95.5	−1.3	0.002	95.5	−1.1	0.006	95.6	−0.7	0.119
	≥120 and < 240	1156	95.1	−1.7	<0.001	95.1	−1.5	0.002	95.3	−1.1	0.042
	≥240	943	94.7	−2.1	<0.001	94.9	−1.7	0.001	95.0	−1.4	0.015
			Ptrend =	<0.001		Ptrend =	0.003		Ptrend =	0.032	

Green tea	<240	1326	95.8	ref		95.6	ref		95.6	ref	
	≥240 and < 480	1423	96.1	0.3	0.536	96.1	0.4	0.360	96.1	0.5	0.295
	≥480 and < 720	1506	95.6	−0.1	0.764	95.8	0.1	0.795	95.8	0.1	0.794
	≥720	1748	95.4	−0.4	0.457	95.4	−0.2	0.637	95.4	−0.3	0.578
			Ptrend =	0.246		Ptrend =	0.316		Ptrend =	0.246	

Oolong tea	0	3723	95.4	ref		95.6	ref		95.6	ref	
	0> and <60	1244	95.8	0.4	0.406	95.8	0.2	0.687	95.8	0.2	0.702
	≥60	1036	96.5	1.0	0.022	95.9	0.3	0.492	95.9	0.3	0.493
			Ptrend =	0.025		Ptrend =	0.513		Ptrend =	0.516	

Black tea	0	4198	95.7	ref		95.7	ref		95.6	ref	
	0> and <60	1165	95.8	0.1	0.867	95.9	0.2	0.699	96.1	0.4	0.318
	≥60	640	95.4	−0.3	0.616	95.4	−0.3	0.629	95.5	−0.1	0.855
			Ptrend =	0.637		Ptrend =	0.664		Ptrend =	0.893	

Fruit juice	0	3884	95.6	ref		95.6	ref		95.6	ref	
	0> and <100	1576	95.7	0.2	0.667	95.7	0.1	0.755	95.8	0.2	0.688
	≥100	543	96.5	0.9	0.108	96.2	0.5	0.351	96.2	0.6	0.322
			Ptrend =	0.115		Ptrend =	0.352		Ptrend =	0.316	

Soft drink	0	4254	95.7	ref		95.7	ref		95.7	ref	
	0> and <100	1173	95.8	0.1	0.835	95.7	0.0	0.996	95.8	0.1	0.813
	≥100	576	95.9	0.2	0.702	95.8	0.2	0.769	95.9	0.2	0.722
			Ptrend =	0.687		Ptrend =	0.781		Ptrend =	0.705	

Water	0	1539	95.2	ref		95.5	ref		95.5	ref	
	0> and <200	1041	95.1	−0.1	0.845	95.2	−0.3	0.617	95.3	−0.2	0.745
	≥200 and < 500	1581	96.1	0.9	0.062	96.1	0.6	0.197	96.1	0.6	0.185
	≥500	1842	96.1	0.9	0.037	95.8	0.3	0.463	95.8	0.3	0.464
			Ptrend =	0.032		Ptrend =	0.442		Ptrend =	0.490	

Model 1: Adjusted for age (continuous) and PHC areas.

Model 2: Model 1 + adjusted for BMI (continuous), smoking status (never, former or current smoker), alcohol intake (non-drinker or tertiles of alcohol intake), family history of diabetes (yes or no), leisure-time physical activity (<1 day/week or ≥ 1 day/week), daily time spent walking (<30 min, 30 min to <1 h, 1 h to <2 h, or ≥2 h), employment status (employed or unemployed) and hypertension (yes or no).

Model 3: Model 2 + adjusted for the daily amount of teaspoons of sugar used in tea or coffee + respective beverage consumption except the variable of interest.

As for the association between the types/amounts of beverages consumed and the HbA1c level (Table 5 and Table 6), analysis using Model 3 showed that coffee and water consumptions were significantly associated with the HbA1c level in the men. Coffee consumption of ≥240 mL/day was associated with a change of the HbA1c level by 0.06% (p = 0.027) as compared to coffee consumption of 0 mL/day. Water consumption of ≥500 mL/day was associated with a change of the HbA1c level by −0.06% (p = 0.022) as compared to water consumption of 0 mL/day. In women, oolong tea and black tea consumptions were significantly associated with the HbA1c levels. Oolong tea consumption of ≥60 mL/day was associated with a change of the HbA1c level by 0.05% (p = 0.005), as compared to oolong tea consumption of 0 mL/day. Black tea consumptions of 1–59 mL/day and ≥60 mL/day were associated with changes of the HbA1c levels by 0.04% (p = 0.016) and 0.04% (p = 0.036), respectively, as compared to black tea consumption of 0 mL/day.

**Table 5:** HbA1c and the types/amounts of beverages consumed in men.

Type of beverage	Daily consumption (mL)	Men (n = 3852)									
		n	Model 1			Model 2			Model 3		
			Adjusted mean	Difference	P-value	Adjusted mean	Difference	P-value	Adjusted mean	Difference	P-value
Coffee	0	1160	5.53	ref		5.54	ref		5.52	ref	
	0> and <120	1072	5.53	0.00	0.917	5.53	0.00	0.904	5.53	0.01	0.760
	≥120 and < 240	765	5.51	−0.02	0.546	5.51	−0.02	0.330	5.52	0.00	0.943
	≥240	855	5.58	0.05	0.065	5.57	0.03	0.188	5.58	0.06	0.027
			Ptrend =	0.046		Ptrend =	0.135		Ptrend =	0.012	

Green tea	<240	1070	5.55	ref		5.54	ref		5.54	ref	
	≥240 and < 480	964	5.51	−0.04	0.130	5.51	−0.03	0.217	5.51	−0.02	0.316
	≥480 and < 720	937	5.55	0.00	0.953	5.55	0.01	0.820	5.55	0.01	0.659
	≥720	881	5.55	0.00	0.918	5.55	0.01	0.718	5.55	0.01	0.566
			Ptrend =	0.515		Ptrend =	0.399		Ptrend =	0.317	
											
Oolong tea	0	2362	5.53	ref		5.54	ref		5.54	ref	
	0> and <60	770	5.53	0.00	0.958	5.53	−0.01	0.635	5.54	0.00	0.899
	≥60	720	5.55	0.02	0.515	5.54	0.00	0.950	5.54	0.00	0.896
			Ptrend =	0.502		Ptrend =	0.994		Ptrend =	0.909	
											
Black tea	0	3047	5.54	ref		5.54	ref		5.54	ref	
	0> and <60	561	5.52	−0.02	0.432	5.52	−0.02	0.353	5.52	−0.02	0.537
	≥60	244	5.57	0.03	0.361	5.56	0.02	0.540	5.57	0.04	0.329
			Ptrend =	0.497		Ptrend =	0.714		Ptrend =	0.404	

Fruit juice	0	2544	5.54	ref		5.54	ref		5.54	ref	
	0> and <100	996	5.52	−0.03	0.196	5.52	−0.03	0.180	5.52	−0.02	0.409
	≥100	312	5.55	0.00	0.968	5.55	0.01	0.859	5.56	0.02	0.629
			Ptrend =	0.654		Ptrend =	0.730		Ptrend =	0.879	

Soft drink	0	2071	5.55	ref		5.55	ref		5.55	ref	
	0> and <100	1057	5.52	−0.03	0.135	5.52	−0.03	0.196	5.52	−0.02	0.320
	≥100	724	5.54	−0.01	0.633	5.53	−0.02	0.523	5.53	−0.01	0.637
			Ptrend =	0.622		Ptrend =	0.515		Ptrend =	0.663	

Water	0	809	5.57	ref		5.57	ref		5.57	ref	
	0> and <200	610	5.57	0.01	0.849	5.58	0.01	0.856	5.58	0.01	0.64
	≥200 and < 500	921	5.52	−0.05	0.053	5.52	−0.06	0.029	5.52	−0.05	0.073
	≥500	1512	5.52	−0.05	0.050	5.51	−0.06	0.012	5.51	−0.06	0.022
			Ptrend =	0.055		Ptrend =	0.011		Ptrend =	0.012	

Model 1: Adjusted for age (continuous) and PHC areas.

Model 2: Model 1 + adjusted for BMI (continuous), smoking status (never, former or current smoker), alcohol intake (non-drinker or tertiles of alcohol intake), family history of diabetes (yes or no), leisure-time physical activity (<1 day/week or ≥ 1 day/week), daily time spent walking (<30 min, 30 min to <1 h, 1 h to <2 h, or ≥2 h), employment status (employed or unemployed) and hypertension (yes or no).

Model 3: Model 2 + adjusted for the daily amount of teaspoons of sugar used in tea or coffee + respective beverage consumption except the variable of interest.

**Table 6:** HbA1c and the types/amounts of beverages consumed in women.

Type of beverage	Daily consumption (mL)	n	Women (n = 6003)								
			Model 1			Model 2			Model 3		
			Adjusted mean	Difference	P-value	Adjusted mean	Difference	P-value	Adjusted mean	Difference	P-value
Coffee	0	1984	5.49	ref		5.49	ref		5.48	ref	
	0> and <120	1920	5.49	−0.01	0.728	5.49	−0.01	0.710	5.50	0.02	0.214
	≥120 and < 240	1156	5.48	−0.01	0.486	5.48	−0.01	0.429	5.49	0.01	0.474
	≥240	943	5.49	0.00	0.989	5.50	0.01	0.792	5.50	0.03	0.198
			Ptrend =	0.987		Ptrend =	0.760		Ptrend =	0.293	

Green tea	<240	1326	5.50	ref		5.50	ref		5.50	ref	
	≥240 and < 480	1423	5.49	0.00	0.810	5.50	0.00	0.925	5.50	0.00	0.891
	≥480 and < 720	1506	5.48	−0.01	0.416	5.48	−0.01	0.455	5.48	−0.01	0.420
	≥720	1748	5.49	−0.01	0.605	5.49	−0.01	0.587	5.49	−0.01	0.485
			Ptrend =	0.638		Ptrend =	0.568		Ptrend =	0.465	

Oolong tea	0	3723	5.47	ref		5.48	ref		5.48	ref	
	0> and <60	1244	5.50	0.03	0.033	5.50	0.02	0.165	5.50	0.02	0.230
	≥60	1036	5.55	0.08	<0.001	5.53	0.05	0.002	5.53	0.05	0.005
			Ptrend =	<0.001		Ptrend =	0.003		Ptrend =	0.006	

Black tea	0	4198	5.48	ref		5.48	ref		5.48	ref	
	0> and <60	1165	5.51	0.03	0.072	5.51	0.03	0.065	5.52	0.04	0.016
	≥60	640	5.52	0.04	0.033	5.52	0.04	0.053	5.52	0.04	0.036
			Ptrend =	0.023		Ptrend =	0.039		Ptrend =	0.029	
										

Fruit juice	0	3884	5.49	ref		5.49	ref		5.49	ref	
	0> and <100	1576	5.49	0.00	0.864	5.49	0.00	0.791	5.49	0.00	0.742
	≥100	543	5.49	0.00	0.970	5.48	−0.01	0.571	5.48	−0.01	0.516
			Ptrend =	0.986		Ptrend =	0.556		Ptrend =	0.499	
											
Soft drink	0	4254	5.50	ref		5.50	ref		5.50	ref	
	0> and <100	1173	5.49	−0.01	0.605	5.48	−0.01	0.422	5.49	−0.01	0.483
	≥100	576	5.46	−0.04	0.095	5.46	−0.04	0.079	5.49	−0.04	0.078
			Ptrend =	0.093		Ptrend =	0.069		Ptrend =	0.073	

Water	0	1539	5.49	ref		5.50	ref		5.50	ref	
	0> and <200	1041	5.46	−0.03	0.127	5.46	−0.03	0.087	5.47	−0.03	0.109
	≥200 and < 500	1581	5.49	0.00	0.790	5.49	0.00	0.944	5.49	0.00	0.934
	≥500	1842	5.51	0.02	0.162	5.50	0.00	0.850	5.50	0.00	0.991
			Ptrend =	0.029		Ptrend =	0.376		Ptrend =	0.512	

Model 1: Adjusted for age (continuous) and PHC areas.

Model 2: Model 1 + adjusted for BMI (continuous), smoking status (never, former or current smoker), alcohol intake (non-drinker or tertiles of alcohol intake), family history of diabetes (yes or no), leisure-time physical activity (<1 day/week or ≥ 1 day/week), daily time spent walking (<30 min, 30 min to <1 h, 1 h to <2 h, or ≥2 h), employment status (employed or unemployed) and hypertension (yes or no).

Model 3: Model 2 + adjusted for the daily amount of teaspoons of sugar used in tea or coffee + respective beverage consumption except the variable of interest.

## 4 Discussion

The present study was conducted to investigate the associations between the types/amounts of beverages consumed and the glycemic measures in the JPHC Diabetes cohort. The associations between the types/amounts of beverages consumed and glucose metabolism have been reported from previous studies [[2], [3], [4], [5], [6], [7], [8], [9], [10], [11], [12], [13], [14], [15], [16],^18^,[23], [24], [25], [26]]. However, assessment of the beverage of choice in daily life seemed complicated and was correlated with the participants’ preferences, characteristics, and lifestyles. In the present study, we comprehensively collected information on the types/amounts of beverage consumed using a questionnaire adapted to the lifestyles of the Japanese population and examined the associations.

The cross-sectional analysis demonstrated that high coffee consumption was significantly associated with a decrease of the FPG level in both men and women. No significant association was observed between consumption of any other type of beverage and the FPG level. On the other hand, significant associations were observed between the HbA1c level and consumptions of several types of beverages. High coffee consumption was associated with an increase of the HbA1c level and high water consumption was associated with a decrease of the HbA1c level in men. High oolong tea and black tea consumptions were associated with increases of the HbA1c level in women.

In regard to the association of glucose metabolism with coffee consumption, a meta-analysis [2] of 28 prospective studies reported the existence of an inverse association between coffee consumption and the risk of type 2 diabetes. In the present study, an inverse association was observed between coffee consumption and the FPG level in both men and women, suggesting that coffee consumption might have a favorable effect on glucose metabolism. However, unexpectedly, a significant positive association was observed between coffee consumption and the HbA1c level in men. The association remained unchanged even after the inclusion of 4625 men who had originally been excluded because their evaluations were performed under non-fasting conditions (Table 7). The conflicting results could be explained by the effect of coffee consumption on the postprandial glucose levels. A meta-analysis [27] reported that short-term trials showed impaired postprandial glucose response after coffee consumption, whereas long-term trials showed improved glucose metabolism. The slight postprandial increases in the glucose levels in those with higher coffee consumption could cumulatively result in an increase of the HbA1c level. The possible mechanism of the postprandial glucose increase could be the caffeine-mediated impairment of glucose uptake in the skeletal muscle [28] via adenosine receptor antagonism [29] or epinephrine release [30], which could alter glycemic index of foods. In addition, before the mid 2000's when sugar-free drinks became popular with improved health consciousness, most canned coffees contained sugar, which could also explain the postprandial glucose increase.

**Table 7:** HbA1c and the types/amounts of beverages consumed in men (including patients evaluated under non-fasting conditions).

Type of beverage	Daily consumption (mL)	n	Men (n = 8477)								
			Model 1			Model 2			Model 3		
			Adjusted mean	Difference	P-value	Adjusted mean	Difference	P-value	Adjusted mean	Difference	P-value
Coffee	0	2571	5.52	ref		5.53	ref		5.51	ref	
	0> and <120	2435	5.54	0.02	0.297	5.54	0.01	0.490	5.54	0.03	0.071
	≥120 and < 240	1617	5.54	0.01	0.558	5.54	0.01	0.764	5.55	0.04	0.051
	≥240	1854	5.58	0.06	0.001	5.58	0.04	0.013	5.59	0.08	<0.001
			Ptrend =	0.001		Ptrend =	0.012		Ptrend =	<0.001	

Green tea	<240	2325	5.55	ref		5.55	ref		5.55	ref	
	≥240 and < 480	2153	5.51	−0.04	0.013	5.51	−0.04	0.033	5.51	−0.03	0.05
	≥480 and < 720	2029	5.56	0.01	0.458	5.56	0.01	0.421	5.56	0.02	0.365
	≥720	1970	5.56	0.01	0.422	5.56	0.01	0.528	5.56	0.01	0.455
			Ptrend =	0.066		Ptrend =	0.127		Ptrend =	0.113	

Oolong tea	0	5339	5.53	ref		5.54	ref		5.56	ref	
	0> and <60	1721	5.54	0.01	0.558	5.54	0.00	0.948	5.54	0.00	0.798
	≥60	1417	5.58	0.05	0.006	5.57	0.03	0.073	5.57	0.03	0.065
			Ptrend =	0.006		Ptrend =	0.067		Ptrend =	0.061	

Black tea	0	6718	5.54	ref		5.54	ref		5.54	ref	
	0> and <60	1240	5.55	0.01	0.424	5.55	0.01	0.582	5.56	0.02	0.283
	≥60	519	5.59	0.05	0.063	5.58	0.04	0.123	5.59	0.05	0.074
			Ptrend =	0.052		Ptrend =	0.113		Ptrend =	0.060	

Fruit juice	0	5517	5.55	ref		5.55	ref		5.55	ref	
	0> and <100	2272	5.54	−0.01	0.422	5.54	−0.01	0.416	5.54	−0.01	0.669
	≥100	688	5.53	−0.02	0.390	5.53	−0.02	0.387	5.53	−0.02	0.463
			Ptrend =	0.298		Ptrend =	0.294		Ptrend =	0.436	

Soft drink	0	4383	5.56	ref		5.56	ref		5.56	ref	
	0> and <100	2411	5.53	−0.04	0.018	5.53	−0.04	0.016	5.53	−0.04	0.018
	≥100	1683	5.52	−0.04	0.019	5.52	−0.04	0.010	5.52	−0.05	0.007
			Ptrend =	0.020		Ptrend =	0.011		Ptrend =	0.009	

Water	0	1745	5.56	ref		5.56	ref		5.56	ref	
	0> and <200	1311	5.55	−0.01	0.792	5.55	−0.01	0.663	5.56	0.00	0.888
	≥200 and < 500	2149	5.52	−0.04	0.050	5.53	−0.04	0.036	5.53	−0.03	0.075
	≥500	3272	5.55	−0.01	0.653	5.54	−0.02	0.236	5.54	−0.02	0.312
			Ptrend =	0.794		Ptrend =	0.571		Ptrend =	0.568	

Model 1: Adjusted for age (continuous) and PHC areas.

Model 2: Model 1 + adjusted for BMI (continuous), smoking status (never, former or current smoker), alcohol intake (non-drinker or tertiles of alcohol intake), family history of diabetes (yes or no), leisure-time physical activity (<1 day/week or ≥ 1 day/week), daily time spent walking (<30 min, 30 min to <1 h, 1 h to <2 h, or ≥2 h), employment status (employed or unemployed) and hypertension (yes or no).

Model 3: Model 2 + adjusted for the daily amount of teaspoons of sugar used in tea or coffee + respective beverage consumption except the variable of interest.

The association between green tea consumption and glucose metabolism remains controversial [[5], [6], [7],^9^,^10^]. A meta-analysis [26] of 17 small-scale trials reported that green tea consumption was associated with significant reductions of the FPG and HbA1c levels. On the other hand, observational studies have found no association between green tea consumption and the plasma glucose or HbA1c level [5,6,8,23,25]. A meta-analysis [31] of randomized controlled trials reported that green tea was associated with a significant reduction of the FPG, but not of the HbA1c level. In the present study also, we did not find any significant association of green tea consumption with the FPG or HbA1c level, lending support to the negative results of previous studies [5,6,8,23,25].

There is still limited evidence in regard to oolong tea and black tea. In one cohort study [12], individuals consuming 1 cup/day or more of black tea had a 14% lower risk of development of type 2 diabetes, although the result was only of borderline statistical significance. A meta-analysis [31] of randomized controlled studies reported that black tea consumption was not associated with any significant decrease of the FPG or HbA1c level, whereas oolong tea consumption was associated with a reduction of the FPG level. Although evidence is scarce, past studies [12,31] have suggested that consumptions of these teas could exert favorable glucose-lowering effects. However, in the present study, no such effect was observed. Instead, female participants consuming 60 mL/day or more of these beverages showed a significant increase of the HbA1c level as compared to those who did not consume them. The results were also confirmed by analysis of a larger sample size (Table 8). As shown in Table 2, age was negatively associated, while alcohol intake was positively correlated with oolong tea and black tea consumptions in women, suggesting that those who consumed oolong tea or black tea were younger and more likely to drink alcohol, implying that their associations with the glucose metabolism could have been influenced by unhealthy lifestyles. In addition, like canned coffees, canned black teas also contained sugar in the earlier era, which could also have exacerbated the postprandial glucose increase and associated increase of the HbA1c level.

**Table 8:** HbA1c and the types/amounts of beverages consumed in women (including patients evaluated under non-fasting conditions).

Type of beverage	Daily consumption (mL)	n	Women (n = 15,193)								
			Model 1			Model 2			Model 3		
			Adjusted mean	Difference	P-value	Adjusted mean	Difference	P-value	Adjusted mean	Difference	P-value
Coffee	0	5064	5.51	ref		5.50	ref		5.49	ref	
	0> and <120	4851	5.50	−0.01	0.51	5.50	−0.01	0.559	5.50	0.01	0.384
	≥120 and < 240	2900	5.49	−0.01	0.236	5.49	−0.01	0.232	5.50	0.01	0.541
	≥240	2378	5.51	0.00	0.946	5.51	0.00	0.729	5.52	0.02	0.077
			Ptrend =	0.983		Ptrend =	0.697		Ptrend =	0.091	

Green tea	<240	3417	5.50	ref		5.50	ref		5.50	ref	
	≥240 and < 480	3678	5.50	0.00	0.954	5.50	0.00	0.766	5.50	0.00	0.819
	≥480 and < 720	3820	5.49	0.00	0.742	5.49	0.00	0.918	5.49	0.00	0.808
	≥720	4278	5.52	0.02	0.115	5.52	0.02	0.073	5.51	0.02	0.113
			Ptrend =	0.054		Ptrend =	0.043		Ptrend =	0.070	
											
Oolong tea	0	9527	5.48	ref		5.49	ref		5.48	ref	
	0> and <60	3191	5.52	0.04	<0.001	5.51	0.02	0.013	5.51	0.03	0.004
	≥60	2475	5.56	0.08	<0.001	5.55	0.06	<0.001	5.55	0.06	<0.001
			Ptrend =	<0.001		Ptrend =	<0.001		Ptrend =	<0.001	

Black tea	0	10,524	5.50	ref		5.50	ref		5.49	ref	
	0> and <60	3111	5.50	0.01	0.404	5.51	0.01	0.28	5.51	0.02	0.113
	≥60	1558	5.53	0.03	0.008	5.53	0.03	0.011	5.53	0.03	0.015
			Ptrend =	0.008		Ptrend =	0.010		Ptrend =	0.012	

Fruit juice	0	9587	5.51	ref		5.51	ref		5.51	ref	
	0> and <100	4283	5.49	−0.02	0.029	5.49	−0.02	0.019	5.49	−0.02	0.019
	≥100	1323	5.49	−0.01	0.337	5.49	−0.02	0.201	5.49	−0.02	0.195
			Ptrend =	0.131		Ptrend =	0.066		Ptrend =	0.075	

Soft drink	0	10,178	5.51	ref		5.51	ref		5.51	ref	
	0> and <100	3297	5.49	−0.02	0.032	5.49	−0.02	0.011	5.49	−0.02	0.031
	≥100	1718	5.47	−0.04	<0.001	5.47	−0.04	0.001	5.47	−0.04	0.001
			Ptrend =	<0.001		Ptrend =	<0.001		Ptrend =	0.001	

Water	0	3714	5.50	ref		5.51	ref		5.51	ref	
	0> and <200	2642	5.48	−0.02	0.089	5.48	−0.03	0.034	5.48	−0.02	0.069
	≥200 and < 500	4074	5.50	0.01	0.583	5.50	0.00	0.789	5.51	0.00	0.979
	≥500	4763	5.52	0.02	0.096	5.51	0.00	0.978	5.50	0.00	0.945
			Ptrend =	0.011		Ptrend =	0.392		Ptrend =	0.516	

Model 1: Adjusted for age (continuous) and PHC areas.

Model 2: Model 1 + adjusted for BMI (continuous), smoking status (never, former or current smoker), alcohol intake (non-drinker or tertiles of alcohol intake), family history of diabetes (yes or no), leisure-time physical activity (<1 day/week or ≥ 1 day/week), daily time spent walking (<30 min, 30 min to <1 h, 1 h to <2 h, or ≥2 h), employment status (employed or unemployed) and hypertension (yes or no).

Model 3: Model 2 + adjusted for the daily amount of teaspoons of sugar used in tea or coffee + respective beverage consumption except the variable of interest.

A recent meta-analysis [3] reported that consumptions of fruit juices and soft drinks were associated with an increase in the risk of type 2 diabetes, and it would seem reasonable to assume that consumption of soft drinks and fruit juices might have unfavorable effects on glucose metabolism. However, no such findings were observed in the present study. More than a half of the participants in this study were categorized as individuals who did not consume any beverage at all. The participants were categorized into three groups according to their beverage consumption levels of 0 mL, 1–99 mL, and 100 mL/day, such that each category include as close a number of participants as possible. Although it was difficult to set an alternative cutoff value, 100 mL/day of beverage consumption might be too small to exert a clinically relevant difference.

There have been conflicting reports regarding the association between plain water consumption and glucose metabolism. One cohort study [14] failed to report any significant association between consumption of water and the risk of diabetes, while others [13,15,16] have reported that high consumption of plain water was associated with a decreased risk of diabetes. The association of glycemic measures with plain water consumption has been rarely reported. The paucity of research might be due to the difficulty in evaluating the independent effects of plain water consumption. As shown in Table 2, plain water consumption was positively correlated with the consumption of all other types of beverages, except green tea. The present study conducted adjustments for the effects of consumption of other types of beverages and found no significant association between plain water consumption and the FPG level. In the analysis of the HbA1c levels, higher water consumption was significantly associated with a decrease of the HbA1c level in men. However, the result could be incidental, because the significant association became attenuated when a larger sample size was analyzed (Table 7).

The present study had several limitations. First, the data on the types/amounts of beverages consumed and lifestyles were collected based on a self-reported questionnaire. Although the validation study reported fair correlations between the dietary records and responses to the questionnaire, misclassification of the information could have influenced the results to some extent. Since such misclassification is considered to be non-differential, the associations observed in the present study could have been underestimated. Second, although we comprehensively collected information on the types/amounts of beverages consumed and lifestyles, there is a possibility that there were unmeasured confounders which could not be controlled in the present study. For example, beverages are consumed during the habit of snacking, which could affect glucose metabolism. Further adjustment for such dietary habits might have led to more accurate assessment. Third, it was difficult to confirm the causal relationship, since the study design was cross-sectional. Further research is required to confirm the causal relationships.

Despite these limitations, there were also many strengths of the present study. It was based on a multicenter population-based cohort across Japan. We comprehensively collected information on the types/amounts of beverages consumed using a questionnaire adapted to the Japanese lifestyle. The laboratory measurements were performed with strict standardization.

In conclusion, we investigated the association between the types/amounts of beverages consumed and measures of the glycemia status in a population-based cohort across Japan. The cross-sectional analysis showed that high coffee consumption was significantly associated with a decrease of the FPG level in both men and women. In regard to the association with the HbA1c levels, high coffee consumption was associated with an increase of the HbA1c level, and high water consumption was associated with a decrease of the HbA1c level in men. High oolong tea and black tea consumptions were associated with an increase of the HbA1c level in women. Some of the results observed were unexpected. In this point, focusing not only on types/amounts of beverages but also components of beverages such as caffeine could offer clues for elucidating the association between beverage consumption and glycemic measures. In this regard, further research is required.

## CRediT authorship contribution statement

**Yusuke Kabeya:** Writing – original draft, Formal analysis. **Atsushi Goto:** Writing – review & editing, Supervision. **Masayuki Kato:** Investigation, Supervision, Methodology. **Yoshihiko Takahashi:** Investigation, Supervision, Methodology. **Akihiro Isogawa:** Investigation, Supervision, Methodology. **Yumi Matsushita:** Investigation, Supervision, Methodology. **Tetsuya Mizoue:** Investigation, Supervision, Methodology. **Manami Inoue:** Investigation, Supervision, Methodology. **Norie Sawada:** Investigation, Supervision, Methodology, Funding acquisition. **Takashi Kadowaki:** Conceptualization, Methodology, Supervision, Funding acquisition. **Shoichiro Tsugane:** Conceptualization, Methodology, Supervision, Funding acquisition. **Mitsuhiko Noda:** Conceptualization, Methodology, Investigation, Supervision, Funding acquisition.

## Declaration of competing interest

There are no competing interests to declare.
